# A Diagnostic Electronic Reporting Framework Proposal Using Preassigned Automated Coded Phrases

**DOI:** 10.5539/gjhs.v5n2p176

**Published:** 2012-01-03

**Authors:** Lamprini Karpouzou, John Mylonakis, Michalis Evripiotis, Evgenia Mainta, Panayiotis Vasileiou

**Affiliations:** 1 Fund of Providence for Private Sector Employees, Athens, Greece; 2 Economist, Athens, Greece; 3 Hellenic Telecommunications Organization, Athens, Greece; 4 Radiologist, Guy's and St Thomas NHS Trust, London, UK; 5 Radiologist, National Healthcare System, Aghios Dimitrios, Greece

**Keywords:** radiology, chest X-rays reports, consult letters, diagnosis, auto-text, radiology information system (RIS)

## Abstract

Radiologists daily diagnose a large number of Chest X-rays and it is crucial that these reports are appropriately recorded, meaningfully indexed, carefully stored, easily retrieved, shared and printed. The absence of organized reports’ storage does not permit their direct and easy retrieval, while after almost a year the report is perished and not even readable (handwritten or typed). The scope of this paper is to evaluate and propose the use of preassigned automated-coded phrases for the chest X-ray electronic reporting in a Radiology Department. The research included 9,252 typed reports, using the proposed method and 949 hand written reports (later typed or not), which were used to compare between the time being spent in reporting with either method. The results proved that even if the method could not be applied fully, there was a 90% reduction of the time being spent by the radiologists and secretarial staff in a Radiology Department, thereby facilitating the typing and management of the electronic archives. In addition, it was found that the reprinting due to addendums/discrepancies, when the proposed method was used, was reduced fourfold, when compared to the previously used methods. In conclusion, the consistent application of preassigned automated-coded reporting can be time saving, cost effective and environmentally friendly saving paper and ink.

## 1. Introduction

Radiologists daily diagnose a large number of Chest X-rays and it is crucial that these reports are appropriately recorded, meaningfully indexed, carefully stored, easily retrieved, shared and printed (Hall & Lemoine, 1986). Information Technology Systems could assist this task by incorporating science information and health care management that interact with resources, i.e. personnel, devices and methods, required to optimize the acquisition, storage, retrieval and communal use of all needed patient information (Karvouni, 2010, Hundt et al., 1998), combining, thereby, low cost and quality improvement methodology (Niemeijer et al., 2012).

In a Radiology Department, Radiologists used and still apply routine manual reporting of X-rays in simple carbonless paper (paper 1 based files), with later filing and storage of the hard copies by the secretarial staff (Eng & Eisner, 2004). The absence of organized reports’ storage does not permit their direct and easy retrieval, while after almost a year the report is perished and not even readable (handwritten or typed). While computerized systems are increasingly being used in Radiology Departments, it is significant to invent and use widely accepted consult letters (coding) in order to speed reports’ completion, their storage potential and capacity and future data manipulation. The scope of this paper is to evaluate the benefits of using preassigned automated-coded phrases for the chest X-ray reporting in a Radiology Department.

## 2. Past Literature

International literature is still limited on the computerized reporting systems and techniques in healthcare, although its magnitude is vast on the general use of Information Science, Health Information Systems and Programs, Web Services and Health Informatics. Similarly, the papers found in Greek publications were very few (Maniatis et al., 1998, 2000), making this paper a pioneer. Overall, past literature revealed limited adoption of this technology into the world medical setting. Therefore, it was decided to present all relevant papers found on the health reporting and messaging technology, regardless of strict Radiology reporting purposes.

Hall & Lemoine (1986) researched the routine manual encoding of pathological data, using the SNOP and SNOMED systems at two London teaching hospitals. They reported that both systems were considerable, efficient and important in coding accuracy. Howard (1986) described a discharge letter introduced into the South and Central Birmingham Geriatric Service, which combined the features of a discharge notification and a discharge summary. The authors concluded that this method finally reduced the workload of the Scientific and Secretarial staff and speeded communication with General Practitioners, for whom it represented an acceptable of the conventional discharge summary.

Marichal et al. (1987) introduced the ‘ARTEMIS’ in France which is a standardized and computerized medical file which intended to improve the follow-up of hypertensive patients, the efficacy of treatment and to comprehend and endorse national-wide surveys. Tessier (1993) found out that medical transcriptions can attest to the accuracy of their transcription but they cannot attest to the accuracy of patient or provider identification. The quality of patient care documentation can only be attested to by provider review and signature (through authentication).

Maniatis et al. (2000) reported that the time needed for full reports’ coding is considerably high, even if one would use the friendliest computer and user interface systems. Lieb et al. (2007) examined the case of consult letters in patients’ system in Germany. They concluded that consult letters are the main way of relaying information between attending physicians involved in patient treatment at different levels of care and should therefore reach continuing care physicians with minimal delay.

Willis & Quigley (2011) reviewed the quality of reporting in published meta-analyses of diagnostic tests, using PRISMA statement and establishing whether there has been a measurable improvement over time. They concluded that although there has been an improvement, there were still many deficiencies in the reporting methodology, which reviewers need to address in order to enhance the validity of the reported findings.

Rao et al. (2012) evaluated the impact of a short messaging system for clinical assessments, telephone calls and delays to surgical drain removal. They concluded that benefits of a short messaging system protocol included reduced number of clinic visits while concerns regarding physician privacy, compensation and time need to be addressed before further application of this technology.

Gurol-Urganci et al. (2012) assessed the effects of mobile phone messaging for communicating results of medical investigations on peoples’ healthcare-seeking behavior and healthcare outcomes. They reported very limited evidence of indeterminate quality, of the communication of medical investigations’ results by mobile phone messaging, which may make little or no difference to women's anxiety overall or in women with positive test results but may reduce anxiety in women with negative test results.

Meyer et al. (2012) assessed the effects of using email for communicating results of diagnostic medical investigations to patients, compared to SMS/text messaging, telephone communication or usual care, on outcomes, including hazards, for health professionals, patients and caregivers and health services. They reported that they could not draw any conclusions on the effects of using email for communicating results of diagnostic medical investigations to patients and thus no recommendations for practice can be stipulated.

## 3. Research Methodology

2005 was the year with the highest number of Chest X-rays carried out in the University Radiology Department of Athens during the last decade. More precisely, during the year 2005, 29,861 X-rays were conducted, out of which 15.264 were Chest X-rays.

A handwritten report was given for 4,037 cases, mostly because of being conducted during on call shifts, while a typed report was given in the remaining 11,227 cases. 1975 X-rays were excluded from the material used since the typed reports were diagnosed by Radiologists that were not part of the research group. For the same reason, a further 3.088 of the 4.037 handwritten reports were also excluded from the material used, leaving a total of 9,252 reports appropriate for use in the research (Table 1).

**Table 1:** Chest X-rays data

15,264 Chest X-rays for year 2005			
**11,227** typed reports of Chest X-rays		**4,037** of hand-written reports of Chest X-rays	
1975 reports made by Radiologists not in the research group	9,252 of Chest X-rays of the Thorax diagnosed by our research group	949 of Chest X-rays were diagnosed by Radiologists of the research group	3,088 of Chest X-rays were diagnosed by Radiologists not in the research group
7,470 reports using only the pre-assigned coded automated phrases	1,003 reports using the preassigned coded automated phrases with complementary comments	779 reports where it was impossible to apply the method	Used as a control group

Overall, reporting using the preassigned coded phrases for the entire report was applied to 7,470 cases. In 1,003 cases, the research group was obligated to use complementary phrases, while in 779 cases the proposed method could not be applied at all. The 949 cases of hand-written reports by the Radiologists of the research group were timed and used as a control group to evaluate the speed of the method.

The materials used by the research group were the following: A PC Pentium III 1,6 MHz (256 Mb Ram), a hard disc HD 60 GB, with the logistics programme Windows XP- service pack 2, Word 2003. All data gathered were saved in the server of the Radiology Department. The reporting was timed with the use of a chronometer. The word count of the reports was done manually for the handwritten reports and with the appropriate Word 2003 tools for the typed reports.

The research group noted the most frequently used phrases in the reports of the Chest X-rays performed in the 2^nd^ semester of 2004. Thus, a list was made with the 58 mostly applied phrases and subsequently grouped by the anatomical compartment they referred to (general, pulmonary parenchyma, hilum-mediastinum, costophrenic and cardiophrenic angles, diaphragm, pleura, heart, thoracic spine-ribs, catheters and recommendations) and were each assigned a code. The codes consisted of 2-3 letters and 1-2 numbers, each of which was assigned an automated phrase. Every phrase could be put in the report by using only its code from the appropriate list. The list of phrases with their corresponding code was distributed to Radiologists with the obligation to use the appropriate code during reporting.

The time consumed for the diagnosis and writing of the report (with and without the use of coded phrases), as well as, for the official hand-written report (without the use of codes) were documented. Furthermore, the procedure of typing was timed from draft, with or without the use of the tools of automated coded phrases. In addition, it was taken into account the time spent when reprinting was needed, due to addendums or discrepancies in the reporting with or without the use the preassigned automated coded phrases, regardless of their etiology (typing mistake, wrong interpretation of the draft). Timing measurement results and the mean times were calculated in seconds (sec) converted by 100 letters. The reprinting due to mistakes is expressed by pages (by 100 letters, Table 2).

**Table 2:** Time results presentation

Procedure	Evaluated variable			

	Mean time for			Reprinting
	Writing	Typing	Total	
1. Writing of the official hand-written report	131	0	131	-
2. Writing and typing of draft report without the tools of preassigned automated coded phrases	108	103	211	0.12
3. Writing of draft report with the use of preassigned automated coded phrases and consequent typing with the tools of preassigned automated coded phrases	9	5	14	0.03

## 4. Research Results

- The original process of handwritten reports (keeping always a copy) was calculated to be 131sec. The 2nd process of a hand-written draft, typing and saving it in electronic archives was calculated to be 211sec (108sec+ 103sec). The overall delay was calculated to be 80sec (211-131 sec).
- In the proposed method, the use of coded phrases (wherever possible) in the writing of the draft report and the consequent typing with the use of the preassigned automated coded phrases and subsequent saving of the report in the electronic archives, demanded 14sec (9sec+ 5sec). In comparison to the original process of giving out hand-written reports, while always keeping a copy (which took 131sec), the time saved was as much as 117 secs (131secs – 14secs).
- The time benefit from typing of reports with the use of the preassigned automated coded phrases instead of not using them was calculated to be 197 secs (211-14sec), always in sec/100 letters. The relative value is 15.01 (211:14sec), which means that the task completion was accelerated by 15 times.
- The reprinting due to addendums or discrepancies with the proposed method is four (4) times less frequent in comparison to the previous used methods.
- The mean number of coded phrases used in each report was 4.1
- The mean number of used letters in each report was 214.1.

The percentage of reports written with the sole use of preassigned automated coded phrases amounted to 80.7%, while the percentage of reports that did not contain any coded phrases amounted to 8.4%. This finding leads to the conclusion that with the use of preassigned automated coded phrases, we benefited:

- 465 hours / man from the writing of the draft
- 453 ¾ hours / man from the typing of the report (in total 918 ¾ hours / man)
- From the printing of reports: 1,494 sheets of paper with the accordingly used ink.

## 5. Research Limitations

For the purpose of this paper, it was assumed that all typists recognize easily the handwriting of Radiologists. The time taken in order to save a report electronically was not mentioned since it was the same for both procedures, regardless of the use of the preassigned automated codes or not. Also, the time taken to diagnose an X-ray is not included in the calculations. Finally, the number of new handwritten reports due to addendums/discrepancies in the previous ones was not included in the results. It was not possible to make codes equivalent to the National Classification of Diseases, although that was not one of the researchers’ goals.

## 6. Conclusions

The scope of this paper was to evaluate and propose the use of preassigned automated-coded phrases for the chest X-ray electronic reporting in a Radiology Department. The research was based on 9,252 typed reports, using the proposed method and 949 hand written reports (later typed) which were used to compare between the time being spent in reporting with either method. Results showed that with the use of preassigned automated coded phrases, Radiologists could benefit 465 hours from the draft writing, 453 ¾ hours from reports typing and 1,494 printed sheets of paper and the used ink.

Overall, the use of preassigned automated coded reporting in a Radiology Department facilitates typing and management of the electronic archives. It is simple, easy to use, adaptable and evolving. The proposed method proved to be time saving and cost effective or even more cost-free to whoever owns a PC (Microsoft Office 2003) with current up to date programming. The method is flexible and allows the existence of different codes according to each Radiologist's preferences. It is always possible to expand the list with new codes without affecting the old ones, as long as the new phrases do not assume the same code letters-numbers. Each Radiologist may form his/her own list with the most frequently used phrases.

Research results cite with past literature studies which are in favor of using preassigned automated-coded phrases in reporting, proving that they reduce the workload in Radiology Departments and speeds communication among their staff members.

It must be noted that it is not obligatory to cover the whole extent of diseases or to mutually exclude codes per group, as happens in more sophisticated systems, such as Radiology Information Systems
